# Brugada syndrome

**DOI:** 10.1186/1750-1172-1-35

**Published:** 2006-09-14

**Authors:** Carlo Napolitano, Silvia G Priori

**Affiliations:** 1 Molecular Cardiology Laboratories, IRCCS, Fondazione Salvatore Maugeri, Via Ferrata 8, 27100 Pavia, Italy

## Abstract

A novel clinical entity characterized by ST segment elevation in right precordial leads (V1 to V3), incomplete or complete right bundle branch block, and susceptibility to ventricular tachyarrhythmia and sudden cardiac death has been described by Brugada *et al*. in 1992. This disease is now frequently called "Brugada syndrome" (BrS). The prevalence of BrS in the general population is unknown. The suggested prevalence ranges from 5/1,000 (Caucasians) to 14/1,000 (Japanese). Syncope, typically occurring at rest or during sleep (in individuals in their third or fourth decades of life) is a common presentation of BrS. In some cases, tachycardia does not terminate spontaneously and it may degenerate into ventricular fibrillation and lead to sudden death. Both sporadic and familial cases have been reported and pedigree analysis suggests an autosomal dominant pattern of inheritance. In approximately 20% of the cases BrS is caused by mutations in the *SCN5A *gene on chromosome 3p21-23, encoding the cardiac sodium channel, a protein involved in the control of myocardial excitability. Since the use of the implantable cardioverter defibrillator (ICD) is the only therapeutic option of proven efficacy for primary and secondary prophylaxis of cardiac arrest, the identification of high-risk subjects is one of the major goals in the clinical decision-making process. Quinidine may be regarded as an adjunctive therapy for patients at higher risk and may reduce the number of cases of ICD shock in patients with multiple recurrences.

## Definition

Brugada syndrome (BrS; OMIM 601144), or "Idiopathic ventricular fibrillation" as defined by some authors [1], is an autosomal dominant form of cardiac arrhythmia, presenting with a typical electrocardiographic (ECG) pattern of ST segment elevation in leads V1 to V3, and incomplete or complete right bundle branch block [2]. Syncope, typically occurring at rest or during sleep, is a common presentation of BrS [3], and it is caused by fast polymorphic ventricular tachycardia. In some cases, tachycardia does not terminate spontaneously. It may degenerate into ventricular fibrillation and lead to sudden death.

## Epidemiology

The incomplete information concerning the genetic bases of BrS prevents a prevalence assessment among the general population. The current estimate is based upon ECG surveys among healthy subjects. These studies have been mostly done in the Asian population and only one has been carried out among Caucasians. The suggested prevalence ranges from 5/1,000 (Caucasians) to 14/1,000 (Japanese) [4-6]. The higher prevalence of the BrS ECG pattern in far eastern countries has been recently confirmed in a large clinical study [7] (see paragraph Genetic bases and pathophysiology). In this region the disease is thought to represent a frequent cause of sudden death among young individuals and is named as: lai-tai, pokkuri, sudden unexplained death syndrome, sudden unexplained nocturnal death syndrome.

## Clinical description

BrS manifests with syncope and cardiac arrest, typically occurring in the third and fourth decade of life, and usually at rest or during sleep. In 1998 Brugada *et al*. presented data on 63 patients in whom, after a mean follow up of 34 ± 32 months, 34% of previously symptomatic (syncope and/or cardiac arrest) patients had recurrence, while a first cardiac event occurred in 27% of the asymptomatic individuals. These results called for an aggressive therapeutic strategy in all patients with BrS and, since no pharmacological treatment of proven efficacy was (and still is) available, it led to implantable cardioverter defibrillator (ICD) implants in several young asymptomatic individuals. However, a different picture is emerging from more recent epidemiological surveys. In 2000, Priori *et al*. showed an incidence of 16% for the recurrence of a cardiac arrest in symptomatic patients, while none of the asymptomatic individuals at enrolment had a cardiac event after three years of follow up [8]. The low incidence of events at follow up in the subgroup of patients who are asymptomatic at diagnosis has been subsequently confirmed by several authors [7,9-12]. It is important to stress the concept that this figure might be biased by the fact that it is currently not known whether a Brugada-like ECG always indicates the presence of the disease or whether it is a non-specific finding in some cases.

### ECG pattern

**Figure 1:**
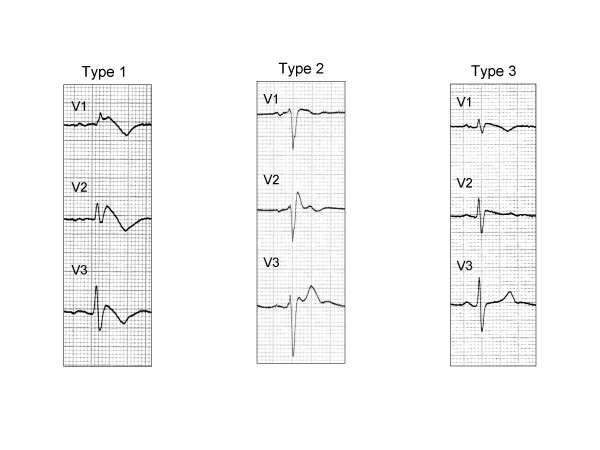
**ECG pattern in Brugada syndrome**. According to recent consensus document (ref 13), type 1 ST segment elevation either spontaneously present or induced with Ajmaline/Flecainide test is considered diagnostic. Type 1 and 2 may lead to suspicion but drug challenge is required for diagnosis. The ECGs in the right and left panels are from the same patient before (right panel, type 1) and after (left panel, type 1) endovenous administration of 1 mg/kg of Ajmaline during 10 minutes.

BrS is characterized by a typical ECG pattern of incomplete or complete right bundle branch block and ST segment elevation (≥ 2 mm) in leads V1 through V3 with a "coved morphology". This pattern may be spontaneously evident or induced by a provocative pharmacological test with sodium channel blockers (Ajmaline or Flecainide). The diagnostic criteria for BrS have been recently updated and summarized in a consensus article [13]. According to this document Brugada syndrome can be established in the presence of:

1) ST-segment elevation (type 1, figure 1) in more than one right precordial lead (V1 to V3), and one of the following: documented ventricular fibrillation; self terminating polymorphic ventricular tachycardia; a family history of sudden cardiac death (<45 years); coved type ECGs in family members; electrophysiological inducibility; syncope. There should be no other factor(s) that can account for the ECG abnormality.

2) Appearance of type 2 ST-segment elevation ("saddle-back type") (figure 1) in more than one right precordial lead upon challenge with a sodium channel blocker. A drug-induced ST-segment elevation to a value >2 mm should raise the possibility of Brugada syndrome when one or more clinical criteria are present (see the case 1 above).

3) Appearance of type 3 ST segment elevation in more than one lead under baseline conditions with conversion to type 1 after challenge with a sodium channel blocker is considered equivalent to case 1 above.

## Genetic bases and pathophysiology

At the present time, only one BrS-related gene is known. Mutations in the cardiac sodium channel gene, *SCN5A*, on chromosome 3p21-23, were identified by Chen in 1998 [14]. Interestingly, BrS is not the only phenotype linked to mutations in this gene. Known allelic disorders are: the LQT3 variant of Long QT syndrome [15] (OMIM 603830), the progressive cardiac conduction defect (PFHB, OMIM 113900) and the Sick sinus syndrome (SSS1; OMIM 608567) [16-18]. *In vitro *expression of mutant SCN5A proteins showed that BrS, PFHB and SSS1 are characterized by a loss of sodium channel function, whereas in LQT3 there is an excess of sodium inward current [14]. To add to the complexity of the *SCN5A*-related phenotypes, overlapping syndromes have been also described in association with some specific *SCN5A *mutations that may cause the coexistence of LQT3 and BrS [19-21] or LQT3, BrS and PFHB in the same individuals [17,22].

Unfortunately, *SCN5A *mutations account for approximately 20% of BrS cases [23]. Only one additional locus (on chromosome 3p22-25 [24]) has been identified by linkage analysis in a single large family, but, despite screening of several candidates in the region, the corresponding gene has not been found.

A possible explanation of the race-specific differential prevalence of the BrS phenotype has been suggested by Bezzina *et al*. [25]. Resequencing of the *SCN5A *promoter region allowed these authors to identify an Asian-specific haplotype: a six single nucleotide polymorphism (SNP) block not present among Caucasians, that is associated with lower transcription activity of the gene. The haplotype has an impressively high frequency of 0.22 in the Asian population and it is clinically associated with a reduced intraventricular conduction velocity. Thus, a constitutively reduced inward sodium current (I_Na_) may be present and favor the high rate of occurrence of the BrS ECG pattern in far east countries.

### Cardiac structural abnormalities in Brugada syndrome Genotype-phenotype relationship

In their initial report on eight patients, Brugada *et al*. emphasized the lack of structural cardiac abnormalities [2]. However, at that time other authors had already reported the typical V1-V3 ST segment elevation and right bundle branch block in some patients with arrhythmogenic right ventricular cardiomyopathy (ARVC; OMIM 107970) [26,27]., thus suggesting a possible overlap between these clinical entities.

More consistent data on a causal relationship between *SCN5A *mutations and structural abnormalities have been reported thereafter. The initial anecdotal cases of dilated cardiomyopathy (DCM) [28] and histological abnormalities [29] in *SCN5A *mutation carriers have been recently confirmed by systematic screening for this gene in DCM probands. These studies identified five allelic variants co-segregating with the phenotype [30,31]. Finally, Frustaci *et al*. reported structural degeneration (fibrosis) and apoptosis during the analysis of myocardial biopsies of patients with a clinical diagnosis of Brugada syndrome and a *SCN5A *mutation [32]. Taken as a whole, these studies strongly suggest that at least some *SCN5A *mutations manifest as both excitability and structural derangement. On a clinical basis, this possibility should always be considered and it advocates the need of careful echo and nuclear magnetic resonance (NMR) studies on all BrS patients.

## The role of molecular diagnosis in BrS

*SCN5A *has a 6.048 Kb coding sequence spanning 28 exons. As of January 2006, approximately 160 *SCN5A *mutations had been reported[33], and approximately 65% of them are associated with a BrS phenotype, but no specific clustering within the coding region is demonstrable. No hot spots have been reported.

As a result of this genetic heterogeneity, the screening for known mutations is not feasible. Therefore, most of the molecular analysis laboratories usually screen the *SCN5A *coding region by performing single strand conformational polymorphism (SSCP) or denaturing high performance liquid chromatography (DHPLC), and DNA sequencing.

Establishing a diagnosis of BrS in an asymptomatic individual based on the ECG phenotype is a big responsibility for the clinician, as it implies informing a young "healthy" subject that they are at risk of sudden death and are at risk of generating affected offspring.

Molecular genetics may free the cardiologist from the burden of defining the diagnosis in difficult cases. This may be particularly important in conditions such as BrS that may present incomplete penetrance [34,35]. In this case, the detection of a genetic defect within a family may represent the only tool for the identification of all those who are at risk of cardiac events and transmitting the disease to their offspring. This information has a direct impact on clinical management. Obviously, the limited yield of genetic testing in BrS does not allow diagnosis to be excluded in negative patients.

Furthermore, with the exception of a tendency for a more severe conduction delay among patients carrying a mutation in *SCN5A *[36], clinical manifestations do not differ between genotyped and not-genotyped patients, thus making genotyping an inappropriate method for risk stratification [23].

## Differential diagnosis

ST segment elevation in leads V1-V3 may be found during acute anterior myocardial infarction. In such instances, angina pectoris and myocardial necrosis markers – creatine kinase (CK), creatine phosphokinase-MB (CPK-MB), Troponin I and lactate dehydrogenase (LDH) – are common findings and the differential diagnosis is easily established. Furthermore, there are several other disorders in which, although not constantly observed, ECG abnormalities resembling BrS may develop: arrhythmogenic right ventricular dysplasia/cardiomyopathy, Prinzmetal's variant angina, acute pericarditis/myocarditis, Friedreich's ataxia, Duchenne muscular dystrophy, hypercalcemia/vitamin D intoxication, hyperkalemia, acute pulmonary thromboembolism, acute cholecystitis, transthoracic cardioversion, myotonic dystrophy type 1, Chagas disease, hypothermia, vomiting. Obviously, all of the above mentioned disorders should be considered in patients with suspected BrS.

Besides specific diseases, before BrS diagnosis can be definitely established, it is important to exclude drug-related causes of BrS-like ECG patterns. All antiarrhythmic drugs with a sodium channel blocking effect, especially class Ia and Ic drugs, must be avoided (asymptomatic BrS patients are often diagnosed incidentally after administration of one of these compounds to treat supraventricular arrhythmias). Other drugs reported to be potentially pro-arrhythmic in Brugada syndrome patients are:

• Local anesthetics (non antiarrhythmic): bupivacaine [37];

• Cocaine[38];

• Alpha adrenergic agonists: methoxamine, noradrenaline [39];

• Beta-blockers: propranolol [39];

• Potassium channel activators: pinacidil [40];

• Parasympathetic agonists: acetylcholine [41];

• Ergot alkaloids: ergonovine [41];

• Tricyclic antidepressants [42];

• Opioid analgesics: propoxyphene [43];

• Lithium [44];

• First-generation antihistamines: dimenhydrinate [45];

• Propofol [46].

## Clinical management and risk stratification

Being affected by a genetically determined disease, BrS patients are exposed to a life-long risk of events, but the disease is usually characterized by very long (years) intervals of complete well-being between the cardiac events. Therefore, the implant of an ICD may remarkably impair quality of life and it is of utmost importance to precisely identify the subgroup of individuals in whom this aggressive therapeutic approach is mandatory because of their high-risk of cardiac events.

Treatment guidelines for Brugada syndrome clearly emphasize that patients who have already experienced a cardiac arrest should receive an ICD [47]. However, these patients are in the minority, since most BrS patients are either asymptomatic or are referred with one or few syncopal spells in their clinical history. Risk stratification in these latter subgroups requires careful evaluation of the clinical presentation. Observational studies [23,48]. on the natural history of BrS in large cohorts concordantly suggest that patients with a history of syncope plus a spontaneously abnormal ECG (*i.e. *independent of the provocative test with intravenous sodium channel blockers) have an approximately 6-fold higher risk of cardiac events (syncope and cardiac arrest) as compared with asymptomatic patients and patients in whom the diagnosis is established only after a provocative test. The presence of only spontaneous ST segment elevation was associated with a moderate risk of life-threatening events (Hazard Ratio 2.1) [49], while a history of syncope alone was not an independent predictor of outcome. These patients, as well as the silent gene carriers, belong to a low risk group. Although rare instances of life-threatening events may occur among this latter category, the most effective strategies for their identification remain to be established and the wide-spread use of ICD is not indicated, since device-related complications outweigh the benefits.

The role of programmed electrical stimulation (PES) as a risk stratification tool in Brugada syndrome is still a matter of debate. While some authors support it [13,50], others have provided experimental evidence suggesting a poor predictive value and low reproducibility [23,51] Several factors have been advocated to explain this discrepancy [52] and two prospective studies (that are likely to provide a definitive answer to this issue) are now ongoing, one in the USA and one in Italy.

### Pharmacological therapy

Quinidine, a non-specific blocker of cardiac transient outward current (I_to_) has been proposed as a gene-specific therapy for BrS. This proposal is based on the idea that the loss of sodium inward current in BrS, tilts the balance between outward and inward currents, with an abnormal shift in the outward direction at the end of phase 1 of the action potential. Since transmural distribution of transient inward current is not uniform, this condition creates a dispersion of repolarization and arrhythmogenic substrate [53]. By blocking repolarizing currents, and specifically I_to_, which is active during the initial phases of the action potential, quinidine could restore the equilibrium [54]. Based on this appealing theory, some authors have attempted a gene-specific therapeutic approach for BrS.

The available clinical data show that quinidine prevents arrhythmia inducibility at PES in up to 76% of BrS patients [55-57] and suggest a positive long-term effect in preventing the occurrence of spontaneous arrhythmias [57]. Although no final proof of effectiveness is available, quinidine may be regarded as an adjunctive therapy for patients at higher risk and may reduce the number of cases of ICD shock in patients with multiple recurrences.
